# The ratio between SARS-CoV-2 RNA viral load and culturable viral titre differs depending on the stage of infection: a case study of household transmission in an adult male

**DOI:** 10.1099/acmi.0.000732.v3

**Published:** 2025-02-17

**Authors:** Michael K. Porter, Alexander Viloria Winnett, Linhui Hao, Natasha Shelby, Jessica A. Reyes, Noah W. Schlenker, Anne E. Romano, Colton Tognazzini, Matthew Feaster, Ying-Ying Goh, Michael Gale Jr, Rustem F. Ismagilov

**Affiliations:** 1 Division of Chemistry and Chemical Engineering, California Institute of Technology, Pasadena, CA 91125, USA; 2 Division of Biology and Biological Engineering, California Institute of Technology, Pasadena, CA 91125, USA; 3 Department of Immunology, University of Washington, Seattle, WA 98109, USA; 4 Pasadena Public Health Department, Pasadena, CA 91125, USA

**Keywords:** culture, incident, infectivity, longitudinal, SARS-CoV-2

## Abstract

Effective public health measures for communicable diseases rely on the ability to identify infectious individuals and prevent transmission from those individuals. For severe acute respiratory syndrome coronavirus 2 (SARS-CoV-2), the presence of replication-competent virus in specimens from an individual is the gold standard for confirming infectiousness. However, viral culture from clinical specimens is difficult and infrequently performed. Instead, infectiousness may be inferred based on the abundance of viral RNA (or viral load) in a specimen, which is more easily assessed. For this reason, understanding the relationship between RNA viral load and infectious viral titre has important implications for public health strategy. In this case report, we quantified incident, longitudinal SARS-CoV-2 viral loads collected from saliva and nasal-swab specimens, and viral titre from nasal-swab specimens. We observed that the relationship between viral load and viral titre decreases by over five orders of magnitude throughout the course of the infection. Our work demonstrates the potential for infectious virus even in specimens with low viral loads collected during the early phases of infection.

## Data Summary

The data underlying the results presented in the study are available at CaltechDATA at https://data.caltech.edu/records/cgf4q-byr92.

## Introduction

Throughout the COVID-19 pandemic, the relationship between the detection of viral RNA and replication-competent virus has been used as guiding evidence for infection-control strategies. For example, studies suggesting that low viral-load specimens are unlikely to have observable replication-competent virus [1] were used to argue that low-analytical-sensitivity antigen tests (which only detect high viral loads [2]) would more specifically identify infectious individuals [3,4]. Additionally, the lack of replication-competent virus in specimens collected more than a week after symptom onset [5,10] was used as evidence to release individuals from isolation, despite persistently detectable viral RNA [11].

Assessment of replication-competent virus in clinical specimens is technically challenging [12] and therefore not routinely performed to determine whether an individual is infectious. Rather, studies that have generated viral-culture data are often applied broadly to guide infection-control strategies [13]. However, the design of such studies influences the data, conclusions and resulting policies.

Many studies that assess the presence of replication-competent virus in specimens from individuals with severe acute respiratory syndrome coronavirus 2 (SARS-CoV-2) infection are primarily cross-sectional, include data from only one specimen type and are biassed towards specimens collected late in the course of infection (e.g. after symptom onset) [4,14,18]. However, during the earliest phase of infection, detecting infected individuals can help reduce subsequent transmission [19,20] and improve clinical outcomes [21]. Few studies report viral loads starting from the incidence of acute SARS-CoV-2 infection [13,22,29], and of these, few report both viral-load and viral-culture data [25,27]. If studies of replication-competent virus during SARS-CoV-2 infection are insufficiently representative of early infection, resulting infection control policies may not be optimally effective.

As part of the Caltech COVID-19 Study [23,24, 30], we attempted to fill this gap by capturing both viral-load and viral-titre measurements longitudinally from the incidence of acute SARS-CoV-2 infection in a subset of participants at risk of becoming infected. Within this subset, one individual was found to have an incident infection with the B.1.243 lineage of SARS-CoV-2 while enrolled and collecting twice-daily specimens, from which we measured both anterior-nares (nasal) swab viral load and viral titre. This participant also collected saliva specimens for viral-load measurements. SARS-CoV-2 *N* gene viral loads and human *RNaseP* marker Cq values in saliva and nasal-swab specimens from this individual (Participant AC) have previously been reported [30]. Here, we provide additional quantifications of SARS-CoV-2 *E* and *RdRp* gene viral loads and viral-titre measurements from this participant’s nasal-swab specimens to investigate the relationship between RNA viral load and infectious virus longitudinally from the incidence of naturally acquired infection.

## Methods

### Participant consent statement

This COVID-19 household transmission study was approved under the California Institute of Technology Institutional Review Board under protocol #20–1026, as previously described [23,30]. All adult participants in this study provided signed informed consent prior to enrolment in the study.

### Study design and specimen collection

Participants began self-collecting saliva and nasal-swab specimens immediately upon receipt of specimen collection materials at enrolment, and then each subsequent morning (immediately after waking) and evening (prior to bed). Participants self-collected anterior-nares nasal swabs in Nest viral transport medium (VTM) (catalogue no. NST-NST-202117; Stellar Scientific, Baltimore, MD) and saliva specimens in the Spectrum SDNA-1000 saliva collection kit (Spectrum Solutions LLC, Draper, UT). Study participants were instructed not to eat, smoke, chew gum or brush their teeth for at least 30 min prior to collection and asked to gently blow their noses before nasal swabbing (four complete rotations with gentle pressure in each nostril) with sterile flocked swabs. Specimens were transported daily by medical courier to the Caltech laboratory for analysis. Additional reagent information is provided in Table S1, available in the online Supplementary Material.

### Nucleic acid extraction, quantification of viral load by reverse-transcription quantitative PCR (RT-qPCR) and viral variant determination

Nucleic-acid extraction was performed as previously described [23]. Conversion from reverse-transcription quantitative PCR (RT-qPCR) Cq to viral load (in copies ml^−1^) was determined via calibration curves, reported for *N* gene previously [23], and built for *E* and *RdRP* genes using standard positive controls (IDT 10006896 and IDT 10006897):

$$E\left[\frac{cp}{uL}\right]=2^{\frac{38.241-Cq}{0.9841}}$$

$$RdRP\left[\frac{cp}{uL}\right]=2^{\frac{39.085-Cq}{0.8981}}$$

Nucleic acids extracted from the seventh saliva and nasal-swab specimens collected by the participant underwent viral sequencing and variant determination as previously described [23].

### Measurement of viral titre

A tissue culture infection dose to infect 50% of test cultures (TCID_50_) assay was performed to measure the viral titre in VTM samples. Briefly, the 500 µl VTM sample was filter-cleaned with a spin column (CLS-8160, Corning). VeroE6 cells ectopically expressing human ACE2 and TMPRSS2 (VeroE6-AT cells; a gift from Dr Barney Graham, National Institutes of Health, Bethesda, MD) were seeded confluent in a 96-well plate. After replacing the seeding medium with 90 µl of assay medium (Dulbecco’s Modified Eagle Media+2% heat-inactivated foetal bovine serum+10 mM HEPES+1% penicillin/streptomycin), 10 µl of filtered VTM sample was added to the first row of the plate as the starting inoculation. Then, tenfold serial dilutions were performed in the second through seventh rows, leaving the eighth row as the negative control. Each sample was tested with five replicates. Cells were fixed with 10% formaldehyde and stained with 1% crystal violet 3 days post-infection. Digital photographs were taken, and cell death, indicated by clear areas in a well, was scored to calculate TCID_50_.

## Results

**Fig. 1.**
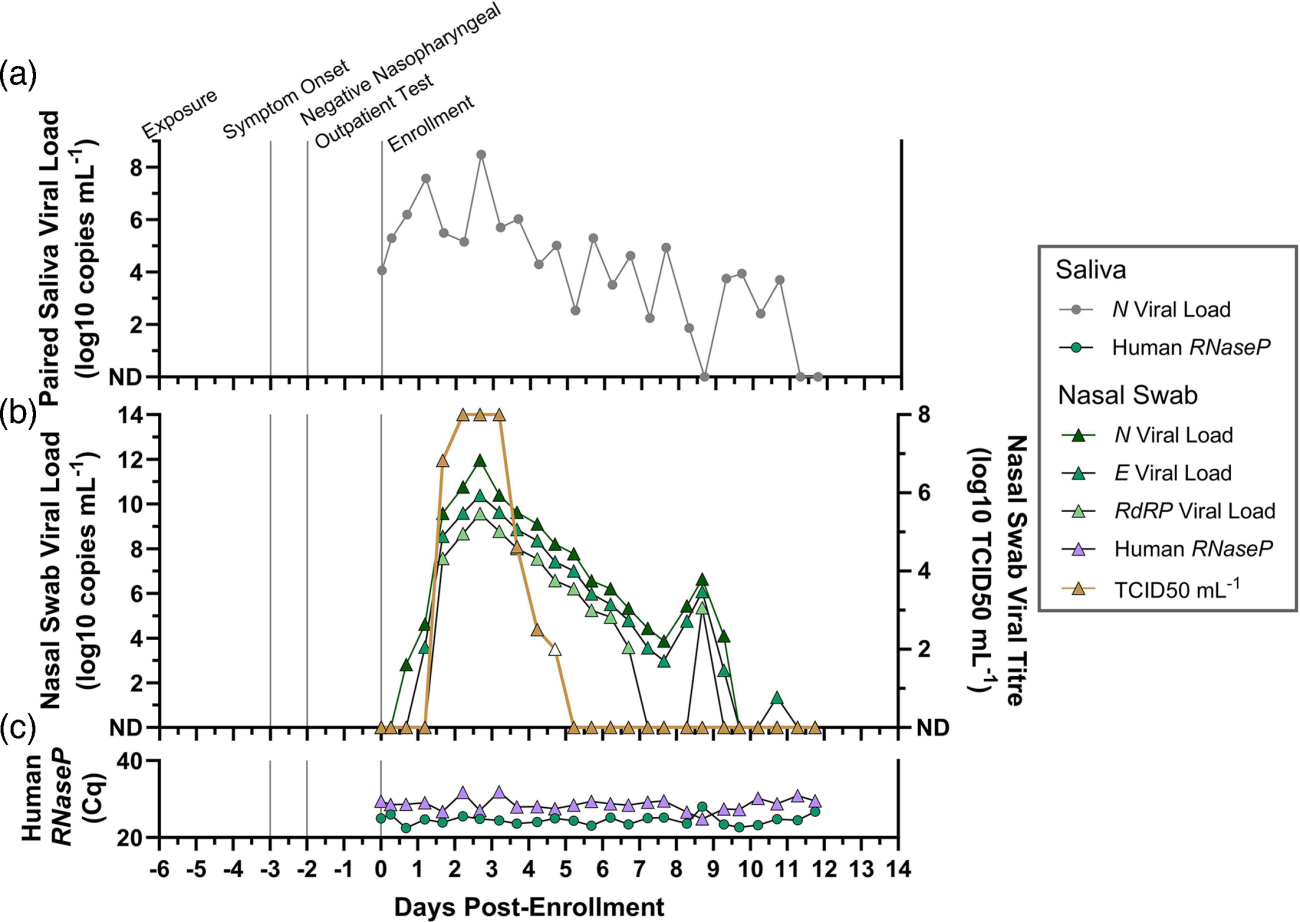
The viral-load and viral-titre trajectories from a single study participant, starting from the incidence of infection. A timeline of Participant AC’s infection is shown with notable case events (exposure, symptom onset, study enrolment), as well as SARS-CoV-2 viral loads in saliva (**a**) and anterior-nares nasal swabs (**b**) on the left *y*-axis, and SARS-CoV-2 viral titre (log_10_ TCID_50_ ml^−1^) on the right *y*-axis. Human *RNase P* Cq values are shown as a measure of sampling consistency and specimen RNA integrity (**c**).

We report the case (Fig. 1) of a 30–39-year-old male (Participant AC), who does not smoke/vape and is otherwise healthy (no chronic medical conditions and self-reported health as ‘very good’). The participant did not report evidence of prior SARS-CoV-2 infection nor receipt of any SARS-CoV-2 vaccine doses. The participant reported taking vitamin C and fish oil supplements, and no other medications. In late January 2021, 6 days prior to enrolment in this study, the participant reported exposure to SARS-CoV-2. Three days prior to enrolment, the participant began experiencing a sore throat, but 2 days prior to enrolment, tested negative on an outpatient, non-rapid nasopharyngeal test. At this time, a household contact of Participant AC (Participant AB, Fig. S1) tested positive, prompting the eligibility of the entire household, including Participant AC, for enrolment in this study.

Upon enrolment, Participant AC had detectable and rising salivary viral loads but was negative in anterior-nares nasal-swab specimens collected over the next day. During this time, the participant remained symptomatic with only a sore throat. On the subsequent day, the participant developed shortness of breath and low (<10^5^copies ml^−1^) nasal viral loads without replication-competent virus detected by culture. After this point, the participant’s nasal-swab specimens achieved high (>10^7^ copies ml^−1^) viral loads and high (>10^6^ TCID50 ml^−1^) viral litres for ~3 days before gradually declining. Throughout this time, headaches, cough, congestion, change in taste/smell, muscle aches and one event of severe nausea were reported, all of which resolved before completion of enrolment.

**Fig. 2.**
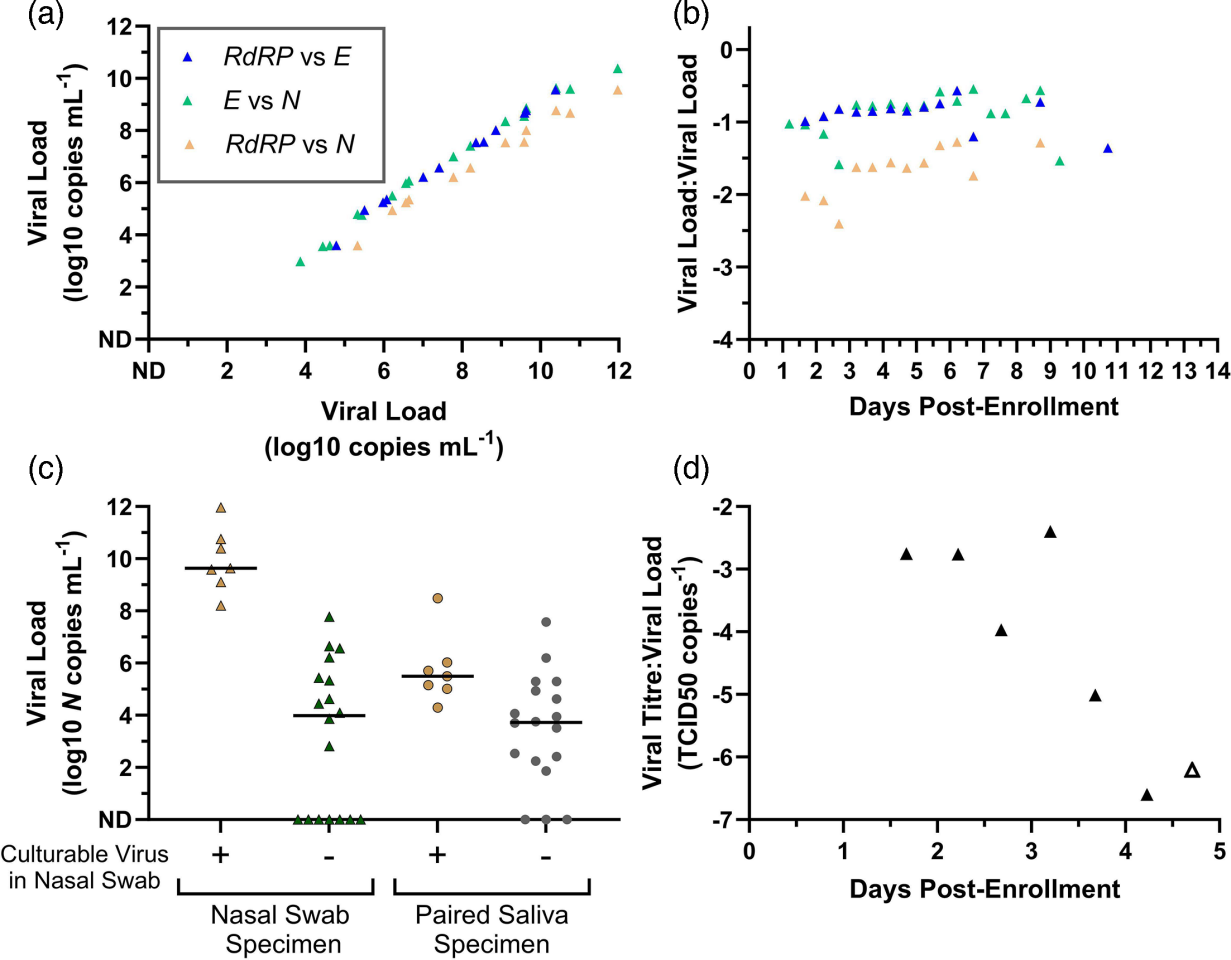
Swab viral loads measured from *N*, *E* and *RdRP* genes remain constant with respect to each other through the course of infection. (**a**) The viral load from one gene is plotted on the *y*-axis with respect to another gene comparing *RdRP* and *E* genes (blue triangles), *E* and *N* genes (green triangles) and *RdRP* and *N* genes (tan triangles). (**b**) The ratios of viral loads are plotted over days post-enrolment for *RdRP* and *E* genes (blue triangles), *E* and *N* genes (green triangles) and *RdRP* and *N* genes (tan triangles). Viral loads that were not detected were omitted from the analysis. nd, not detected. (**c**) Cross-sectional relationship of SARS-CoV-2 viral load (log_10_ *N* copies ml^−1^, *y*-axis) in nasal-swab specimens (triangles) or saliva specimens (circles) based on whether viral-culture positivity (yellow) of the nasal swab from the same time point. The black horizontal bars indicate the median viral load. (**d**) For specimens with detectable viral titre and viral load, the ratio of viral titre (TCID_50_ ml^−1^) over *N* gene viral load (copies ml^−1^) in nasal-swab specimens collected by the participant is plotted through days of enrolment. The open symbol indicates a specimen with detectable but not quantifiable viral titre, for which 100 TCID_50_ ml^−1^ was imputed. nd, not detected.

Cross-sectional SARS-CoV-2 viral loads from different gene targets in nasal-swab specimens correlated closely with each other (Figs1b  2a), and the relationship between viral loads from different gene targets remained proportional throughout the course of infection (Fig. 2b). Cross-sectional analysis of viral load and viral titre revealed that only high viral-load nasal-swab specimens (>10^8^* N* cp ml^−1^) would contain replication-competent virus (Fig. 2c). Additionally, saliva viral load is less distinguishable between samples with and without replication-competent virus in nasal-swab specimens (Fig. 2c). However, longitudinal analysis revealed that the ratio of nasal-swab viral load and viral titre changed by over five orders of magnitude throughout the course of acute infection (Fig. 2d). This relationship indicates that RNA viral load alone, without considering infection stage, may not represent whether a specimen or a person is likely to be infectious or not.

## Discussion

This study quantified viral load and viral titre longitudinally from specimens prospectively collected twice daily by an individual who became infected with the B.1.243 lineage of SARS-CoV-2 through household transmission. High-frequency nasal-swab and saliva sampling from the very beginning of infection and paired measurements of viral load and viral titre in nasal-swab specimens, although challenging to obtain and thus rare, revealed four key findings with implications for infection prevention and control.

First, saliva exhibited higher *N* gene viral loads than in nasal swabs for approximately the first 2 days of incident infection, after which nasal-swab viral loads rose and remained subsequently higher than saliva viral loads. This supports previous observations that SARS-CoV-2 often presents first in oral specimen types before anterior-nares swabs [23,24] and that testing a single specimen type (e.g. nasal swabs) may yield false-negative results during early infection.

Second, replication-competent virus was observed in nasal swabs at many time points when saliva viral loads were low. This suggests that the low viral load of one specimen type is not necessarily indicative of the absence of replication-competent virus in another specimen type.

Third, nasal-swab viral-load measurements from different gene targets (*N*, *E* and *RdRP* genes) correlated strongly with each other longitudinally, such that measurement of any one viral RNA target was indicative of other viral RNA targets [31].

Fourth, we note that the ratio between RNA viral load and culturable viral titre in nasal swabs decreased substantially (greater than five orders of magnitude) through the first week of infection. Cross-sectional analyses of data from Participant AC and in other studies [4,15, 18, 25, 32] have suggested a correlation between viral load and the presence of infectious virus. However, these cross-sectional analyses overlook that the relationship between viral load and infectious virus is dynamic and that early viral loads are more indicative of viral titre than viral loads later in the infection. Therefore, earlier in the infection, individuals with lower viral loads could actually be more infectious than expected based on cross-sectional data.

Data from a SARS-CoV-2 human challenge study [25] supported these conclusions (Fig. S2). In that study, 36 human participants were inoculated intranasally with 10 TCID_50_ viruses, and 18 participants had subsequent sustained detectable infections. We reanalysed longitudinal nasal-swab viral-load and viral-culture data graciously provided by the study authors to compare with what was observed in Participant AC’s naturally acquired infection. Indeed, among specimens with replication-competent virus, the average ratio between viral titre and viral load at each time point after inoculation decreased by nearly four orders of magnitude in the 5 days following inoculation. A later reanalysis of 12 participants in this human challenge study further supported these observations [33].

Taken together, these results caution against conclusions about infectiousness that assume a constant ratio of RNA viral load and culturable viral titre, as commonly inferred based on cross-sectional data or from single specimen types [4,34,36]. Assuming a constant ratio of RNA viral load and culturable viral titre may not reflect early infection nor all anatomical sites from which transmissible virus can be shed and, therefore, may be suboptimal evidence for public health policies that seek to reduce transmission.

We acknowledge three main limitations. First, data are from a single unvaccinated person with acute SARS-CoV-2 B.1.243 infection, acquired prior to the availability of COVID-19 vaccines and the emergence of currently circulating variants. Infection characteristics may exhibit substantial person-to-person variation, and vaccination status and/or viral variant may affect the relationship between viral load and viral titre [37,38]. Second, Participant AC collected saliva specimens in a preservation buffer that precluded the ability to perform viral culture, thereby prohibiting inferences on the relationship between saliva viral load and viral titre, or saliva viral titre and nasal viral titre. Third, the lack of detection of replication-competent virus by viral culture may not reflect a true absence of replication-competent virus in the specimen or shedding of infectious virus by the individual because specimen collection, handling and storage affect virion viability [39,40]. Moreover, both the methods of attempted viral culture and viral characteristics can affect the analytical sensitivity to detect replication-competent virus [41]. Therefore, it is possible that replication-competent virus was present in the first two nasal-swab specimens with detectable viral RNA collected by this participant, but at a concentration below the limit of detection by viral culture.

The data presented here are rare and challenging to obtain. We hope that similar datasets of viral load and viral titre in paired specimen types collected longitudinally starting from early infection can be made accessible for metanalysis and guide optimized public health strategies that reduce the burden of SARS-CoV-2 or other pathogens.

## supplementary material

10.1099/acmi.0.000732.v3 Uncited Supplementary Material 1.

## Abbreviations

RT-qPCR: reverse-transcription quantitative PCR
VTM: viral transport medium
