# Supplementary material for: The ratio between SARS-CoV-2 RNA viral load and culturable viral titre differs depending on the stage of infection: a case study of household transmission in an adult male
Source: Access Microbiol. 2025 Feb 17;7(2):000732.v3. doi: 10.1099/acmi.0.000732.v3 (PMC11833051; doi:10.1099/acmi.0.000732.v3)
Supplement: Uncited Supplementary Material 1. [file acmi-7-00732-s001.pdf]

Supplemental information for:

The ratio between SARS-CoV-2 RNA viral load and culturable viral titer differs depending on stage of infection: case study of household transmission in an adult male

Michael K. Porter[1], Alexander Viloria Winnett[1], Linhui Hao[2], Natasha Shelby[1], Jessica A. Reyes[1], Noah W. Schlenker[1], Anna E. Romano[1], Colton Tognazzini[3], Matthew Feaster[3], Ying-Ying Goh[3], Michael Gale, Jr.[2], Rustem F. Ismagilov[1]

1. Division of Chemistry and Chemical Engineering, California Institute of Technology, Pasadena CA 91125
2. Department of Immunology, University of Washington, Seattle WA
3. Pasadena Public Health Department, Pasadena CA 91125

Contents

Table S1

Figures S1-S2

Detailed Author Contribution Statements

Table S1. Reagent List. Table includes all reagents utilized in this study.

Step	Reagent Name	Description	Manufacturer	Catalogue Number
Specimen Collection	Spectrum SDNA1000 Saliva Collection Device	For at-home collection of spit saliva into a guandinium-thiocyanate based preservation buffer	Spectrum Solutions LLC	SDNA1000
Specimen Collection	NEST Scientific 10mL Sterile Screw-Cap Transport Tube with 3mL VTM	For at-home collection of nasal swab specimens into media that maintains live virions	Stellar Scientific	NST-NST-202117
Nucleic Acid Extraction	MagMAX™ Viral/Pathogen Nucleic Acid Isolation Kit	For extraction of nucleic acids from clinical upper respiratory specimens	ThermoFisher Scientific	A42352
Viral Load Quantification	TaqPath™ COVID-19 Combo Kit	For RT-qPCR measurement of human RNaseP and SARS-CoV-2 N gene	ThermoFisher Scientific	A47814
Viral Load Quantification	Heat-inactivated SARS-CoV-2 2019-nCoV/USA-WA1/2020	Extraction control and standard for RT-qPCR quantification	BEI	NR-52286
Viral Load Quantification	2019-nCoV_E Positive Control	Standard for RT-qPCR quantification	IDT	10006896
Viral Load Quantification	2019-nCoV_RdRp (ORF1ab) Positive Control	Standard for RT-qPCR quantification	IDT	10006897
Viral Load Quantification	E_Sarbeco_F1 Forward Primer, 50 nmol	Forward primer for RT-qPCR measurement of SARS-CoV-2 E gene	IDT	10006888
Viral Load Quantification	E_Sarbeco_R2 Reverse Primer, 50 nmol	Reverse primer for RT-qPCR measurement of SARS-CoV-2 E gene	IDT	10006890
Viral Load Quantification	E_Sarbeco_P1 (FAM) Probe, 25 nmol	Probe for RT-qPCR measurement of SARS-CoV-2 E gene	IDT	10006892
Viral Load Quantification	RdRP_SARSr_F2 Forward Primer, 50 nmol	Forward primer for RT-qPCR measurement of SARS-CoV-2 RdRp gene	IDT	10006860
Viral Load Quantification	RdRP_SARSr_R1 Reverse Primer, 50 nmol	Reverse primer for RT-qPCR measurement of SARS-CoV-2 RdRp gene	IDT	10006881
Viral Load Quantification	RdRP_SARSr_P2 (SUN) Probe, 25 nmol	Probe for RT-qPCR measurement of SARS-CoV-2 RdRp gene	IDT	10007063
Viral Culture	Culture Media	DMEM<br>2%FBS (heat inactivated)<br>1% Penicillin-Streptomycin<br>1% HEPES (1M)	Fisher Scientific<br>Fisher Scientific	MT10013CV<br>SH30071.03<br>MT30002CI<br>MT25060CI
Viral Culture	Cell Line	VeroE6-AT	A gift from Dr. Barney Graham (NIH)	
Viral Culture	Stain for Readout	1% crystal violet<br>20% Ethanol	Sigma-Aldrich<br>Fisher	C-6158<br>4355222

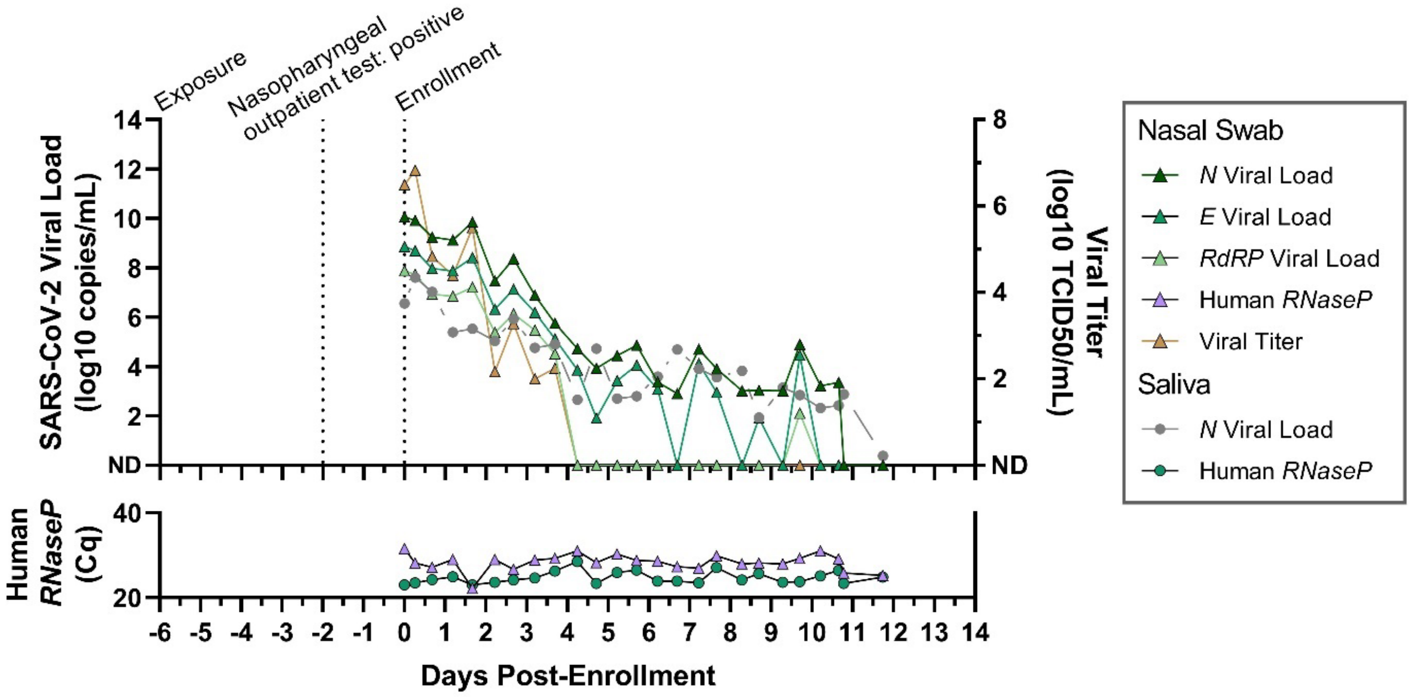

Figure S1. The viral load and viral titer trajectories from a single study participant from the incidence of infection. (A) A timeline of this participant's infection is shown with notable case events (e.g. exposure, positive nasopharyngeal outpatient test, study enrollment), as well as SARS-CoV-2 viral loads (log10 copies/mL) in saliva (circles) and anterior nares nasal swab (triangles) on the left y-axis, and SARS-CoV-2 viral titer (log10 TCID50/mL) on the right y-axis. Human *RNaseP* Cq values are shown as a measure of sampling consistency and specimen RNA integrity. ND, not detected.

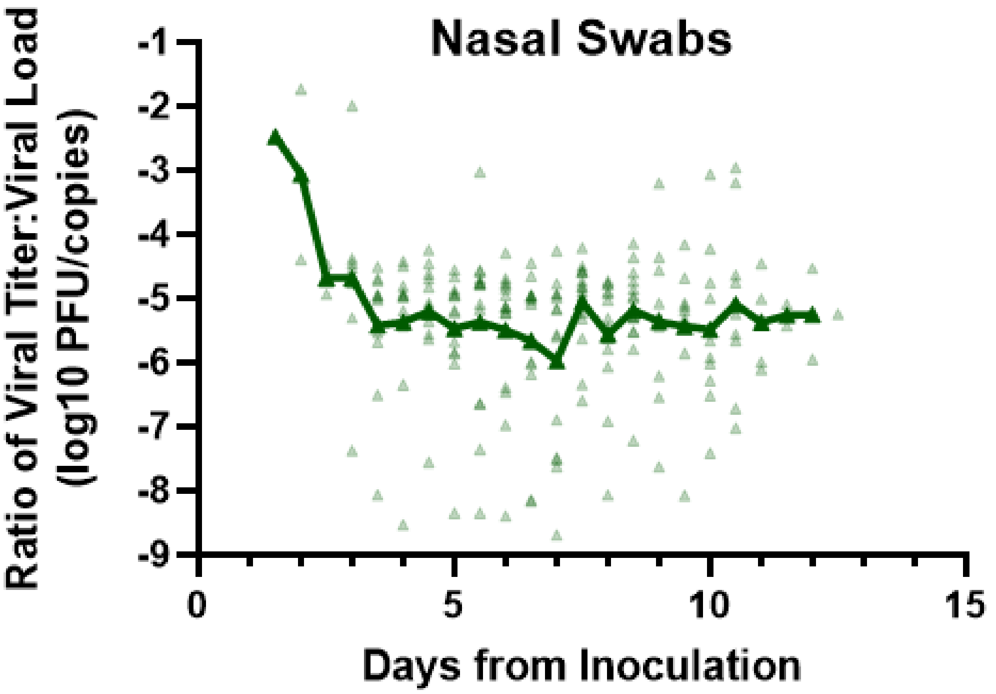

Figure S2. Longitudinal Ratio of Viral Titer to Viral Load from Participants in SARS-CoV-2 Human Challenge Study. As part of a SARS-CoV-2 human challenge study performed in [1] participants were inoculated intranasally with 10 $TCID_{50}$ virus. 18 participants had subsequent sustained detectable infection in nasal swab and throat swab specimens collected daily after inoculation. Viral load and viral culture data from these specimens was graciously provided by the authors of this study. We plotted the log10 transformed ratio of viral titer to viral load in nasal swabs, for all specimens with replication competent virus, by the time from inoculation (green triangles). Green line represents the average log10 transformed ratio of viral titer to viral load among culture-positive nasal swab specimen, for each day following inoculation.

1. Killingley, B., et al., *Safety, tolerability and viral kinetics during SARS-CoV-2 human challenge in young adults.* Nature Medicine, 2022. **28**(5): p. 1031-1041.

Detailed Author Contribution Statements

- Michael K. Porter (MKP)- Conceptualization of nested study with AVW and RFI. Literature review with AVW. Co-wrote biosafety protocols for receipt and processing of live specimens with AVW. Reagent and supply acquisition. Determined and validated live specimen collection methods with AVW. Received, logged and aliquoted live specimens with AVW. Established academic collaboration with colleagues at University of Washington. Performed nucleic acid extractions and *RNaseP, N, E, and RdRP* gene RT-qPCR. Coordinated and prepared shipments of live specimens to collaborators. Interpreted paired viral load and culture data with AVW and LH. Outlined and co-wrote manuscript with AVW. Prepared Figures 1, S1, S2, and S3. Validated underlying data with AVW. Managed author citations and references.
- Alexander Viloria Winnett (AVW)- Conceptualization of nested study with MKP and RFI. Literature review with MKP. Co-wrote biosafety protocols for receipt and processing of live specimens with MKP. Reagent and supply acquisition. Determined and validated live specimen collection methods with MKP. Received, logged and aliquoted live specimens with MKP. Performed nucleic acid extractions and *RNaseP* and *N* gene RT-qPCR. Coordinated and prepared shipments of nucleic acids for viral sequencing. Coordinated and prepared shipments of live specimens to collaborators for viral culture. Interpreted paired viral load and culture data with MKP and LH. Outlined and co-wrote manuscript with MKP. Provided feedback on the design of Figures 1, S1, and S2. Validated underlying data with MKP.
- Linhui Hao (LH)- Received live specimens for viral culture. Performed viral culture at BSL3 and analyzed viral culture data. Interpreted paired viral load and culture data with AVW and MKP. Wrote viral culture methods section. Edited the manuscript.
- Natasha Shelby (NS)- Study Administrator; collaborated on study design and recruitment strategies; created instructions for live viral-load sampling with JAR; enrolled and maintained study participants with JAR and NS. Edited the manuscript.
- Jessica A. Reyes (JAR)- Lead Study Coordinator; created instructions for live viral-load sampling with NS; enrolled and maintained study participants with NS and NWS.
- Noah W. Schlenker (NWS)- Study Coordinator; enrolled and maintained study participants with NS and JAR.
- Anna E. Romano (AER)- Analyzed and summarized viral sequencing data.
- Colton Tognazzini (CT)- Coordinated the recruitment efforts at PPHD with case investigators and contact tracers and provided guidance and expertise on SARS-CoV-2 epidemiology and local trends.
- Matthew Feaster (MF)- Co-investigator; collaborated on study design and recruitment strategies; provided guidance and expertise on SARS-CoV-2 epidemiology and local trends.
- Ying-Ying Goh (YYG)- Co-investigator; collaborated on study design and recruitment strategies; provided guidance and expertise on SARS-CoV-2 epidemiology and local trends.
- Michael Gale (MG)- Co-investigator; coordinated and oversaw viral culture method development and execution. Edited the manuscript.
- Rustem F. Ismagilov (RFI)- Co-investigator; collaborated on study design and recruitment strategies; provided leadership, technical guidance, oversight, and was responsible for obtaining funding for the study.
